# Dissolution of Calcite in the Twilight Zone: Bacterial Control of Dissolution of Sinking Planktonic Carbonates Is Unlikely

**DOI:** 10.1371/journal.pone.0026404

**Published:** 2011-11-15

**Authors:** Andrew Bissett, Thomas R. Neu, Dirk de Beer

**Affiliations:** 1 Max Planck Institute for Marine Microbiology, Bremen, Germany; 2 Helmholtz Centre for Environmental Research – UFZ, Magdeburg, Germany; University of California Merced, United States of America

## Abstract

We investigated the ability of bacterial communities to colonize and dissolve two biogenic carbonates (Foraminifera and oyster shells). Bacterial carbonate dissolution in the upper water column is postulated to be driven by metabolic activity of bacteria directly colonising carbonate surfaces and the subsequent development of acidic microenvironments. We employed a combination of microsensor measurements, scanning electron microscopy (SEM), confocal laser scanning microscopy (CLSM) and image analysis and molecular documentation of colonising bacteria to monitor microbial processes and document changes in shell surface topography. Bacterial communities rapidly colonised shell surfaces, forming dense biofilms with extracellular polymeric substance (EPS) deposits. Despite this, we found no evidence of bacterially mediated carbonate dissolution. Dissolution was not indicated by Ca^2+^ microprofiles, nor was changes in shell surface structure related to the presence of colonizing bacteria. Given the short time (days) settling carbonate material is actually in the twilight zone (500–1000 m), it is highly unlikely that microbial metabolic activity on directly colonised shells plays a significant role in dissolving settling carbonates in the shallow ocean.

## Introduction

Calcareous plankton produces calcium carbonate in the photic zone, which is then transported to deeper regions as sinking detritus. Despite the fact that the shallow ocean is supersaturated with respect to calcium carbonate and that chemical dissolution is theoretically thought to occur only below the aragonite and calcite compensation horizons, it has been reported that dissolution in shallow water (500–1000 m) is common and may play a significant role in determining the amount of carbonate sequestered to sediments.

This discussion was lead by the work of Milliman and co-workers [1], [2], who suggested that shallow water dissolution was common and important, in that it implies a shorter timescale for calcium carbonate cycling within the ocean and has, therefore, implications for possible feedbacks between the marine carbonate cycle and climate. Milliman et al. [2] review the results of past studies that indicate possible shallow water dissolution, attributing their findings to several observations, including a discrepancy between estimates of global pelagic carbonate production, alkalinity budgets and particulate flux data, discrepancies between calcareous plankton standing stocks and fluxes and the fact that certain species of carbonate producers are underestimated in sediment trap flux studies. Finally, Milliman et al. [2] propose several mechanisms for this possible shallow water dissolution; dissolution in the guts of grazers and dissolution mediated by bacterial activity.

Several subsequent modeling studies addressing the quantitative data presented by Milliman et al. [2] have either supported (e.g., [3]) or not supported [4] shallow, water-column, dissolution of calcium carbonates. A discussion of these findings is beyond the scope of this paper, but [4] contains a thorough discussion of Milliman et al.'s [2] findings, and concludes that the concept presented by Milliman et al. [2] and Troy et al. [5] was not correctly and accurately quantified. Indeed, the occurrence of shallow water dissolution is still intensely debated (e.g. Gehlen et al. [6], Doney et al. [7]).

Our intention was, therefore, to investigate the qualitative observations of Milliman et al. [2], Troy et al. [5] and later Schiebel et al., [8], which showed physical changes to carbonates exposed in the shallow ocean, by addressing the suggested mechanism behind the dissolution: bacterially mediated corrosive microenvironments. We, therefore, did not investigate the occurrence of shallow water dissolution per se, but addressed the major suggested mechanism behind this controversial phenomenon.

We conducted experiments specifically designed to test the ability of bacterial colonization and metabolism to create micro-environments conducive to dissolution of shells, thereby, for the first time, addressing experimentally the major mechanism suggested for the mediation of shallow water pelagic dissolution: that specialized microbial consortia colonize the shells, degrade the organic matrix and thereby acidify the shell surface leading to dissolution. We used high resolution microsensor, confocal laser scanning microscopy (CLSM) and scanning electron microscopy (SEM) techniques to track potential biogenic carbonate dissolution, and used molecular methods to test whether indeed specialized consortia colonize the shells.

## Methods

We conducted a series of experiments, each one increasing in complexity, to investigate the possible role of bacteria in dissolution of biogenic carbonate using foraminifera and oyster shells as model dissolution surfaces. Experiments are described chronologically and specific methods for analytical techniques follow:

### Experiment 1 – dissolution and microflora of fresh foraminifera remains

For dissolution studies, 25 benthic foraminifera were collected from shallow (<5 m) Wadden sea sediments (Neuharlingersiel, July 2005), washed 5 times in 50 ml 0.2 µm filtered seawater, sterilized by UV Radiation and incubated in 200 ml ambient seawater at room temperature (approximately 23°C), spiked with 0.5 g sediment, for 5 weeks. Seawater was changed regularly (approx. weekly) to ensure stable pH (8.2). pH (measured on the NBS scale and referred to as pH throughout) was monitored with an MA130 ion detector (Mettler Toledo, Columbus, OH). Experiments were conducted in the lab in open containers, so the carbonate system was in equilibrium with the atmosphere. O_2_, pH and Ca^2+^ microsensors were used to monitor respiratory activity and calcium concentration profiles at the shell/water interface. Foraminifera were destructively sampled and fixed for SEM by immersion for 2 hours in 3% formaldehyde in seawater followed by air-drying throughout the experiment.

For the microbial analysis, 10 foraminifera were washed 5 times in 50 ml 0.2 µm filtered seawater. The washing solution from the final wash was kept and DNA was extracted from both the washed foraminifera and from the final wash solution (50 mL) using the QBIOGENE Fast DNA spin kit for soil, according to manufacturer's instructions. Polymerase chain reaction (PCR) was performed on the DNA extracts using the GM3 and GM4 bacterial primers [9]. PCR products were purified using the QIAquick PCR purification kit (Diagen, Düsseldorf, Germany) and were cloned using the TOPO TA Cloning Kit (Invitrogen, Karlsruhe, Germany) according to the manufacturer's instructions. The clones obtained were screened for the presence of inserts and the positive clones were sequenced with an ABI PRISM 3100 genetic analyzer (applied Biosystems, Foster City, Calif.). The final washing solution was used as a control to check the efficiency of the washing process, which was conducted to remove all loosely attached/unattached bacterial from the foraminifera. No PCR products were generated from this control. Sequences have been deposited at NCBI genebank under numbers JN870229 - JN870278.

### Experiment 2a – Dissolution of remnant foraminifera remains

After the completion of experiment 1, we chose to use larger (approximately 500 µm), remnant (dead remains, shell only) benthic foraminifera obtained from the Red Sea in an attempt to overcome resolution limits imposed by using small shells that may have compromised experiment 1. Remnant foraminifera shells are abundant and easy to collect, allowing us to not only work with larger individuals, but also to more meaningfully replicate our study. Foraminifera shells were sterilized by gamma irradiation and incubated in artificial seawater (ASW) (salinity = 34, pH = 8.2, Alkalinity = 2.4 mEq/L, hw-Meersalz professional, Wiegandt GmbH) under several experimental conditions comprising: sterile/non-sterile, high nutrients/low nutrients combinations. Incubations were made in deep Petri dishes (100 ml). Low nutrient incubations were made in ASW only and high nutrient incubations in ASW+2.5 g L^−1^ peptone and 0.5 g L^−1^ yeast extract (Oxoid, Germany). All incubations were conducted at pH 8.2. The ASW used in these experiments had a calcium carbonate saturation state of approximately 4 [10]. For sterile incubations, the media were autoclaved and the petri-dishes kept closed until sampling. Inoculated incubations comprised autoclaved media spiked with fresh north-sea sediment prior to distribution to petri dishes. Five shells were taken randomly for each treatment and monitored over 4 weeks for dissolution by microscopy (CLSM and SEM) and Ca^2+^ microsensor. At each time point (0, 2, 4, 7, 14, 21, 28 days) Ca^2+^ concentration profiles were measured and 3 replicate foraminifera were removed for EM and 2 for CLSM.

### Experiment 2b - Dissolution of fresh foraminifera remains

In parallel with experiment 2a, 11 living benthic foraminifera, obtained from cultures maintained at the Max Planck Institute for Marine Microbiology, Bremen, were killed and immediately incubated under a subset of the experimental conditions. They were incubated in the sterile and non-sterile high nutrient treatments. Sampling of fresh foraminifera was conducted for SEM only, at the same time intervals indicated above, but without replication.

### Experiment 3 – Dissolution of Oyster shells

The extremely high natural variation in shell architecture, the resultant difficulty in visually detecting small changes in this architecture in destructively sampled shells and the difficulty in obtaining live foraminifera shells led us to use oyster shells as a biogenic carbonate model. They were used in our final experiment, in addition to fresh foraminifera. Oyster shells were sourced from live oysters from the Bremen fish market and were sourced from oyster farms in Normandy, France. Preliminary testing showed that we were able to see differences with CLSM in shell surface topography when both the organic structure and the mineral phase were selectively dissolved with bleach and gluteraldehyde [11] respectively (Figure S1). Oyster shell material was obtained by opening *Crassostrea gigas* oysters, and removing the upper (non-cupped) valve. The adductor muscle was removed; the upper shell washed with sterile ASW and then air dried. Shells were then cut into approximately 1 cm squares using a band saw and the inner surface used as the dissolution surface.

This experiment was designed to assess the potential of both direct bacterial colonization and of bacterial exudates to dissolve carbonate shells. Again sterile and non-sterile incubations were used, including an incubation in media from the non-sterile incubation, after removal of microbes by 0.2 µm filtration. This extra treatment ensured shells were exposed to bulk media chemical changes induced by bacterial metabolism and to bacterial exudates and exo-enzymes, but were not directly colonised. Samples were initially loaded into high nutrient medium, to allow rapid colonization of surfaces, however, after colonization incubation water was replaced with ASW (2 days after inoculation), to reduce the water column bacterial load. CLSM was used to image all shells prior to the experiment's start and throughout the experiment. Samples were taken destructively for SEM at the completion of the experiment. Throughout the incubation Ca^2+^ concentration profiles were taken. Incubation bulk water parameters (temp, pH) were measured daily and maintained at 24°C and pH 8.2.

### Scanning Electron Microscopy

Samples for SEM were air dried, sputter coated with gold, and examined with a Philips Scanning Electron Microscope at 20 kV.

### Microelectrodes

Liquid membrane Ca^2+^ and pH microsensors were prepared and calibrated as described previously [12], [13]. Detection limits for Ca^2+^ microsensors were in the nanomolar range [14]. O_2_ microsensors were prepared as described previously [15]. All electrodes were placed at the shell surface while viewing the sample through a dissection microscope. The surface was then set at 0 µ, and all measurements were made above the surface. Sensors were connected to a micromanipulator, which was fixed to a motorized stage (VT-150, Micos, Eschbach, Germany) and allowed reproducible positioning of the sensor tip with 1 µm precision. The microelectrodes were connected to a picoammeter (O_2_ electrode) or a milivoltmeter, and the meter output was collected by a data acquisition device (NI-Daq 6015, National Instruments, Austin, Texas, USA). After positioning at the surface, profiling was done automatically. Motor control and data acquisition were performed with a computer and custom written software (μ-Profile, Dr. L. Polerecky).

### Laser Scanning Microscopy (CSLM)

CLSM was performed either with a TCS SP MP attached to an upright microscope (Leica, Heidelberg, Germany) or a Zeiss upright LSM 510 microscope (Carl Zeiss, Jena, Germany), both controlled by manufacturers software. Images were collected with 20×0.5 NA, 63×0.9 NA and 63×1.2 NA water-immersible lenses (Leica) or Zeiss Achroplan 40×0.80 W and Zeiss WPlan Apochromat 63×1.0 VIS-IR water immersible lenses in the Z direction for subsequent image analyses. Images were presented as multichannel, maximum-intensity projections using the microscope software. Nonspecific nucleic acid staining was carried out using Syto 9, and SYBR green (Molecular Probes Inc., Eugene, OR, USA). Glycoconjugates in the extracellular polymeric substances (EPS) matrix were stained with Alexa-488 (Molecular Probes) fluorescently labeled *Aleuria aurantia* lectin (Vector, Burlingame, CA, USA) as described previously [16], [17].

### Image analysis

Images were analysed using the Zeiss topography reconstruction software, using the following parameters set: “fill holes”, “fit∶cylinder”. This allowed the assessment of shell surface changes. We first performed an experiment (Figures S1,S2,S3) using chemically treated oyster shells to ensure we were able to detect and distinguish between different types of shell degradation with our method. To imitate selective degradation of the mineral phase of the shells, shells were immersed in 25% gluteraldehyde solution, which both fixes proteins and decalcifies carbonate minerals [11]. To imitate selective degradation of the shells' organic matrix they were immersed for 2 hours in 12% sodium hypochlorite solution [11]. We were able to detect and distinguish between the destructive effects of both treatments using the surface topography reconstruction method described herein. Roughness parameters were measured both over the whole image and as 5 profiles across each image at pixel line numbers 73, 175, 290, 332 and 458, on both raw images and on images filtered to highlight high frequency changes with a Gaussian highpass filter. All roughness parameters measured by the software were analysed. The CLSM images are shown as an iso-surface projection using Imaris (Bitplane Switzerland).

### Statistical Analysis

Statistical analyses were conducted on the CLSM topography data from experiment 3. The experiment was set up as a two-way (3×5) factorial design, with factors treatment (3 levels, inoculated with and without bacteria and control) and time (5 time points). Data not meeting the homogeneity of variances assumption of ANOVA were log transformed. Differences in treatments were assessed for each roughness parameter by factorial ANOVA and a significant effect of the experiment on shell condition was defined as a significant treatment×time interaction term in the model. Significant effects of the main terms in this model do not indicate deterioration of the shells throughout the trial. Principle components analysis (PCA) was also used to visualize the full multivariate data set, using all roughness parameters. Analyses were completed using SPSS10.0 and Primer 5.0 software respectively. All significance tests were performed at α = 0.05.

## Results

### Experiment 1, using small Waddensea foraminifera

**Figure 1:**
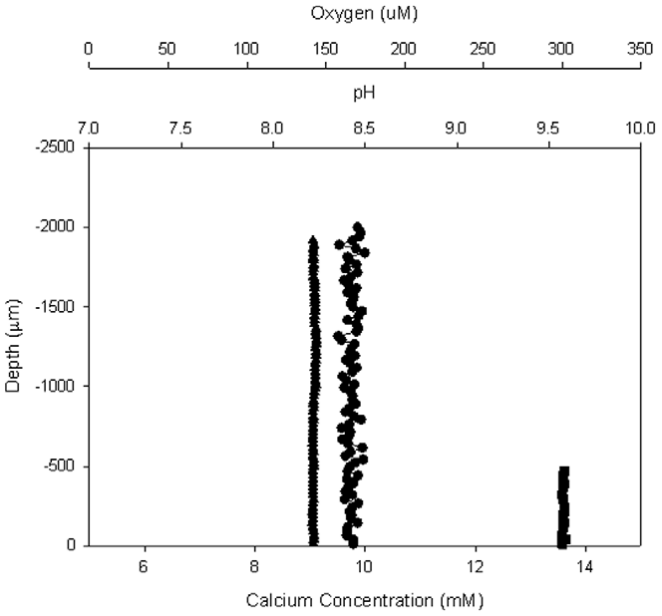
Microsensor profiles. Representative Ca^2+^ (•), O_2_ (▪), and pH (▴) microsensor profiles above bacterially colonised shell. 0 µm indicates the shell surface. Negative distances indicate the microsensor is above the shell, in the overlying water.

Microelectrode concentration profiles did not show any changes in chemical concentrations (Ca^2+^, O_2_, H^+^) at any time or at any point on the foraminifera surface. The lack of change in Ca^2+^ indicated that neither calcium dissolution nor precipitation was proceeding (Fig. 1). The absence of pH and O_2_ gradients indicated low metabolic activity. SEM analysis over 50 foraminifera failed to show any dissolution patterns associated with bacterial colonisation. The microbial consortia associated with the foraminifera were not dominated by a specific strain. Fifty 16S rDNA gene sequences were obtained from the washed foraminifera and the sequences grouped with common marine bacteria. Clones clustered with *Comamonas*, *Vibrio*, *Arcobacter*, *Marinonomas* and *Microbacterium* Spp. (Table 1), all of which are capable of oxygenic respiration and fast growth.

**Table 1:** Number of 16rRNA clones affiliating with each genus, according to RDP Bayesian classifier [28].

Genus	Number of sequences
***Microbacterium***	1
***defluvibacter***	2
***Sulfitobacter***	1
***Arcobacter***	1
***Vibiro***	1
***Marinomonas***	5
***Pseudoalteromonas***	3
***Comamonas***	36

### Experiment 2, using large Red Sea foraminifera shells

**Figure 2:**
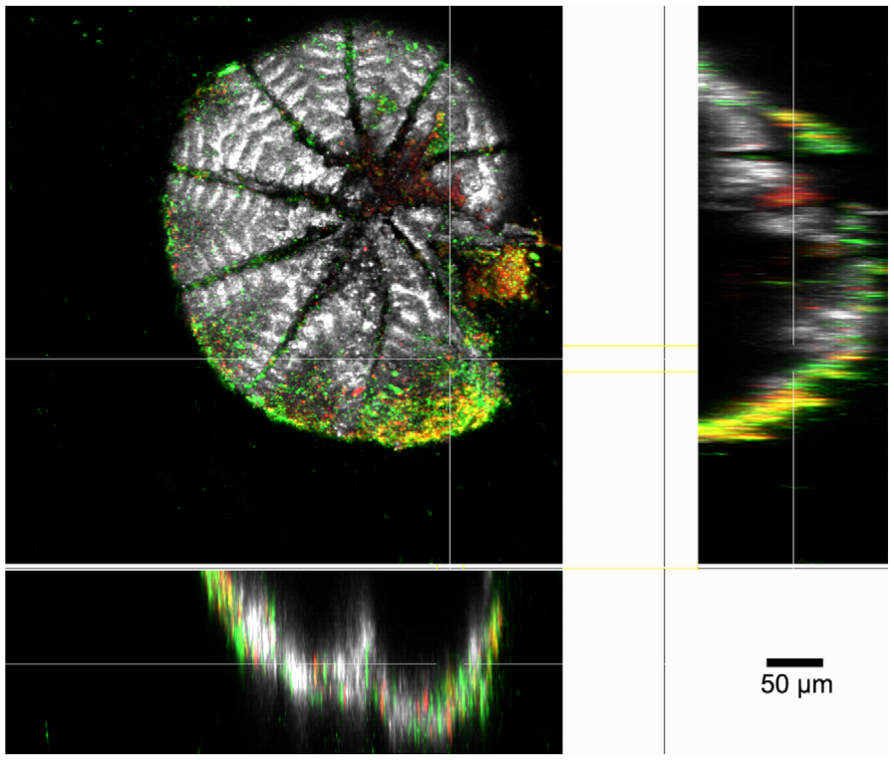
CLSM image of foraminifera shell incubated in non-sterile, dissolution experiment. Bacteria (green) can clearly be seen colonising the shell. Also visible are lectin-stained EPS glycoconjugates (red). Orange/yellow (overlay of green and red) denotes colocalized signal by SYTO9 nucleic acid stain and *Aleuria aurantia* lectin stain. A) Maximum intensity projection of the whole image series. B) Extended XYZ projection of the same data set. The elongated signals in axial direction (XZ and YZ) are due to the low numerical aperture lens used for imaging the whole foraminifera shell. Bar size: 50 µm.

**Figure 3:**
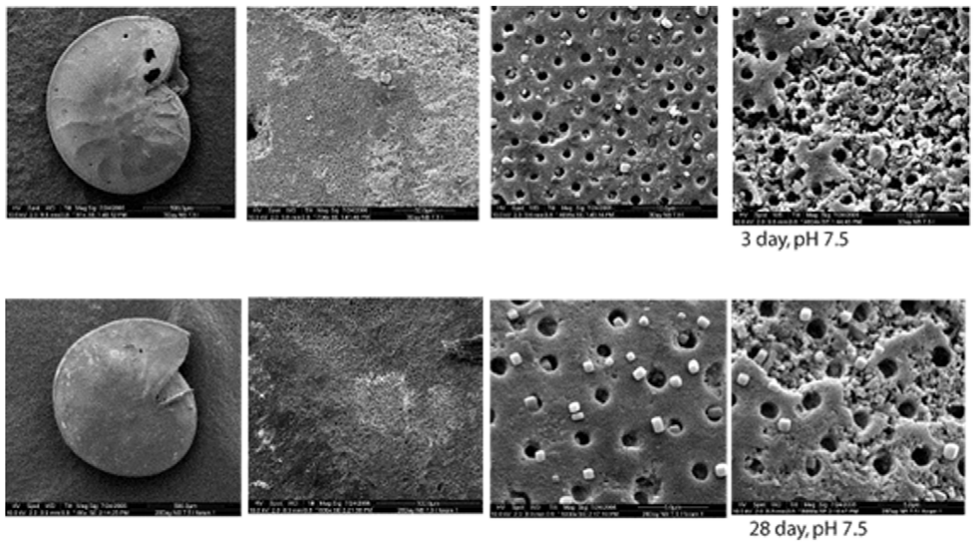
Representative electron micrographs of foraminifera shells incubated in dissolution experiment 2 (low nutrient treatment). Upper row shows one foraminifera after 3 days incubation. Whole animal is represented in the left hand panel, the other 3 panels represent various random positions on the shell. The lower row represents similar pictures of a shell incubated in the same treatment for 28 days. Note the heterogeneity in shell condition. Both shells show areas of shell degradation, and areas of no degradation. Similar results were observed for all shells at all treatment levels.

**Figure 4:**
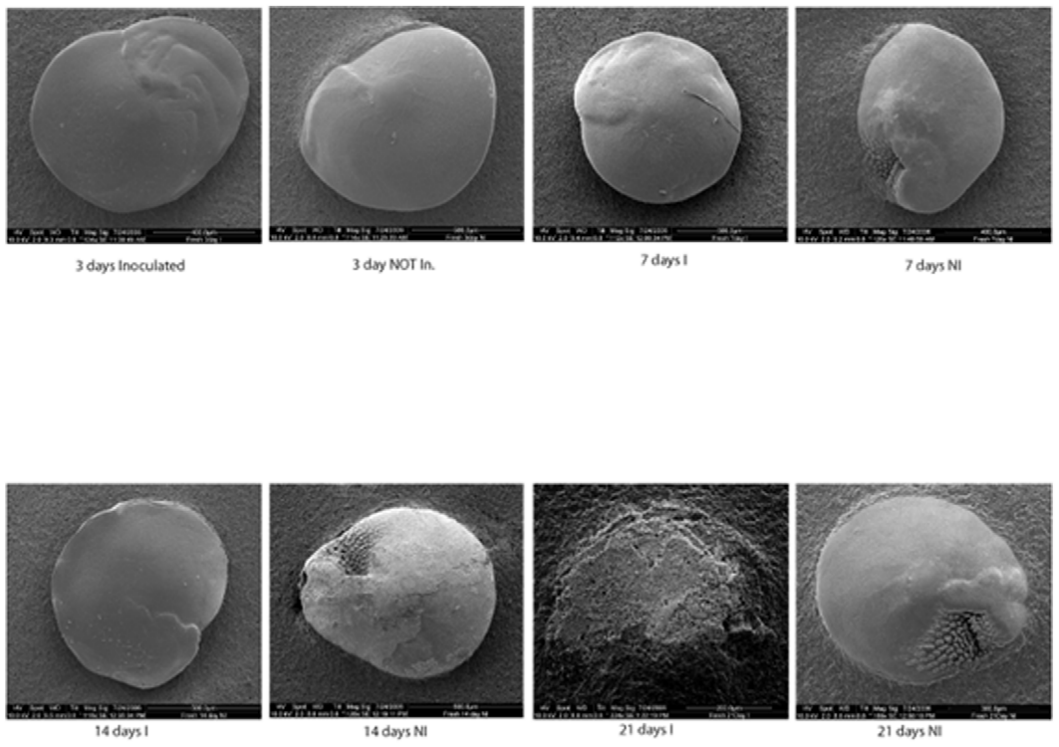
Representative electron micrographs of foraminifera shells incubated in inoculated (I) and uninoculated (NI) treatments described above. Note that over the initial 14 days the NI incubated samples show more deterioration than the I samples. This trend is suddenly reversed in the 21 sample. Only one sample per time could be obtained.

Again no microbially induced dissolution was found. Bacteria rapidly colonized all non-sterile shells (both in low and high nutrient conditions), building biofilms of up to 50 µm thick with abundant EPS (fig. 2). Bacteria were also seen to eventually colonize all sterile treatment shells by the end of the 3^rd^ week. Despite the rapid colonization of inoculated samples, no Ca^2+^ efflux was detected with microsensors. Remnant shells were so inherently heterogeneous that no dissolution patterns attributable to any treatment could be seen by SEM (Figure 3). Interestingly, shells in experiment 2b, fresh large foraminifera shells, showed apparent shell degradation, although no dissolution was observed from microprofiles (Figure 4). Remarkably, more shell degradation was observed in the sterile treatments, than in the inoculated treatments. Bacteria were not observed to colonise this uninoculated treatment. With only one individual per sample point and the obvious degradation of the inoculated sample at the final time point no real trend could be discerned. However, given the lack of bacterially association with shell degradation we could not demonstrate microbially induced corrosion.

### Experiment 3 - Dissolution of Oyster shells

**Figure 5:**
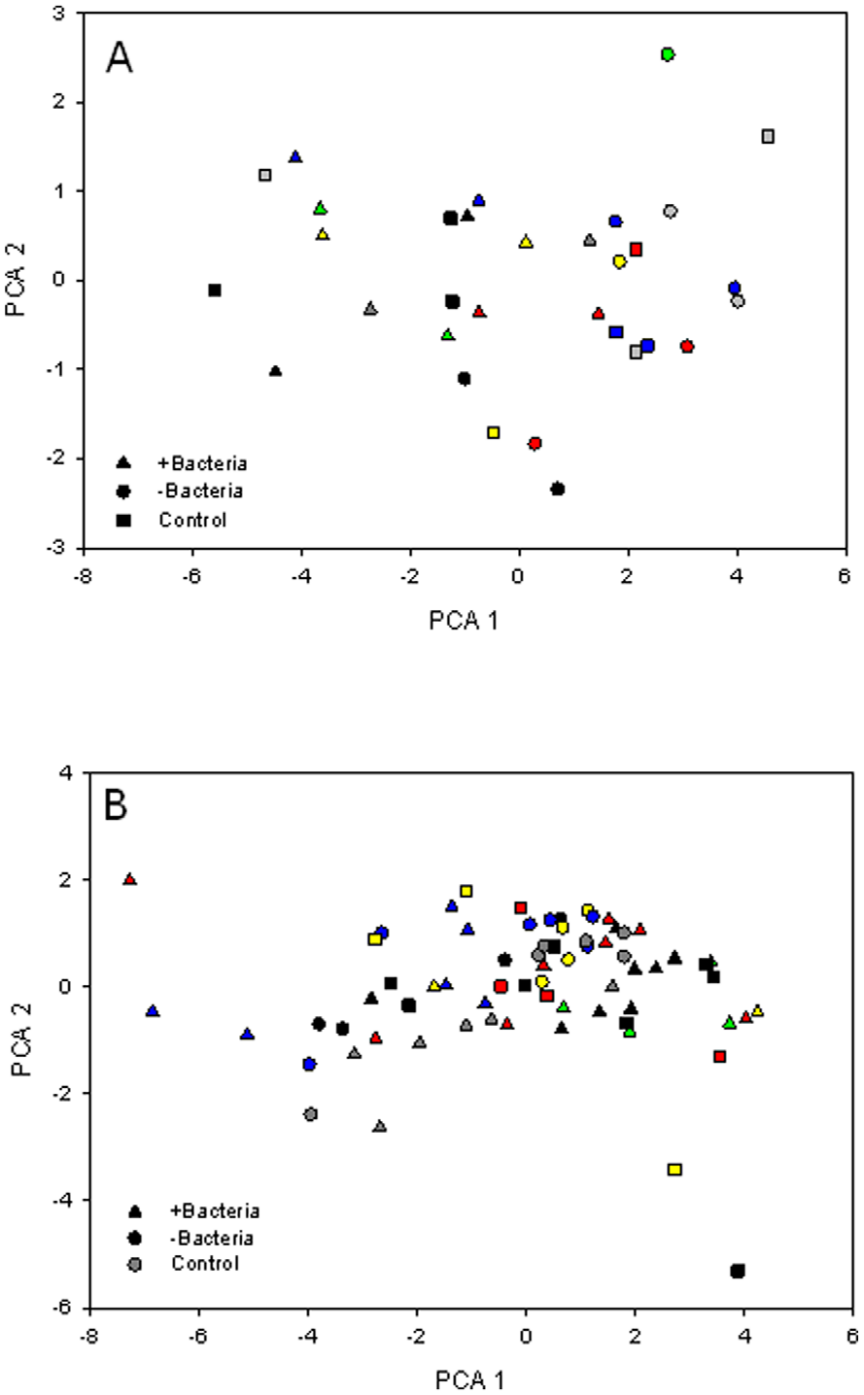
Roughness parameters of carbonate surfaces. PCA plots of CLSM determined shell roughness parameters for: (A) raw oyster shell scans and (B) raw foraminifera shell scans. No groupings of shells based on treatments or times are visible, nor were any treatment or time effects statistically significant (p>0.05). Input data for the plots shown comprises raw (not Gaussian filtered) whole image averaged roughness parameters. Specific pixel line determined data (at line numbers 73, 175, 290, 332 and 458) and Gaussian filtered data are not shown, but returned similar results (i.e., no relationship between bacterial presence and shell roughness (p>0.05)). Treatments represented are shells colonised directly by bacteria (+Bacteria), shells exposed to bacterial culture media (−Bacteria) and control shells. Colors refer to sampling times after the incubation's beginning; black = T0, red = T1, green = T2, yellow = T3, blue = T4, grey = T5.

After the first two experiments, which indicated that direct bacterially induced shell dissolution was unlikely, we sought to control experimental parameters more tightly and monitor the progression of single shells through the entire incubation process. We again measured no effect of the presence of bacteria (either directly through colonization or indirectly via filtered medium) on Ca^2+^ concentration profiles. At the end of the experiment a new piece of shell was added to the system and acid added to the sea-water to stimulate dissolution. The pH was lowered to a value of 7.1, at which time an increase in Ca^2+^ concentration was seen at the shell surface, thus our failure to observe dissolution was not an experimental artifact (Figure S4). CLSM topography roughness data were analysed with univariate and multivariate methods. Changes to the shell surfaces could not be related to the presence or absence of bacteria, because all ANOVA tests returned non-significant interaction terms (time×presence/absence of bacteria, F_9, 15_, p>0.05) for both oyster and foraminifera shells. All data were also included as multivariate inputs and analysed by PCA, but no effect of bacteria on changes to shell roughness over time was observed (figure 5). Finally, SEM images showed changes to the architecture of all shells, but no direct correlation between changes to shell surface and presence or absence of colonizing bacteria.

## Discussion

Experiments demonstrated rapid bacterial colonisation of shell surfaces. We were able to detect Ca^2+^ fluxes from shells when we added acid to induce dissolution (Figure S4) and were able to visualise changes to shell surface topography after selective chemical dissolution of shells (Figures S1,S2,S3). However, we did not detect dissolution in any of our bacterial experiments, neither with microsensors nor with microscopy techniques.

Our experiments were conducted primarily in ASW, with a pH of 8.2 and alkalinity of 2.4 mEq/L and in equilibrium with atmospheric CO_2_, and Ω = 4. These conditions are not identical to that of the twilight zone, which has a pH approx. 7.8 [18] and an Ω around 1.5. It has, however, been suggested that an Ω of <0.8 is needed for dissolution to occur [2]. This being the case for dissolution to occur via metabolically produced CO_2_, a significant pH shift would have had to have occurred, both in our experimental set-up and in the twilight zone. The higher Ω experienced in our system may have resulted in no discernable CaCO_3_ dissolution, but if a microenvironment of sufficient magnitude to induce dissolution (even in the twilight zone) had developed a pH gradient would have been evident. Therefore, while we acknowledge that our experimental conditions were only a rough approximation of those in the twilight zone, our inferences regarding possible processes explaining carbonate dissolution in the twilight zone are valid.

In experiment 2 bacteria were observed in the sterile treatments after 3 weeks of incubation. Although these samples were ideally to remain sterile, the presence of low numbers of bacteria did not necessarily negate their usefulness. The main reason for sterilisation was to prevent the buildup of bacterial communities that could potentially create dissolving microenvironments. Bacteria in sterile treatments were only observed as single cells, with no significant EPS formation, therefore, the prevention of significant bacterial growth was achieved. Thus, despite contamination of these treatments, their utility in discerning dissolution caused by heavy colonisation and the development of acid microenvironments was not compromised.

It has been reported that high concentrations of phosphate may inhibit calcite dissolution [19] and that dissolved organic substrates may have a similar effect. It should, therefore, be noted that the addition of yeast extract to our high nutrient incubations may have resulted in calcite dissolution inhibition in this treatment. The high nutrient treatment was employed only to ensure rapid microbial growth and high nutrient media replaced was by low nutrient media shortly after inoculation (within 2 days). Colonisation occurred in both low and high nutrient media, although more quickly in the latter, and dissolution was not observed in either. The final phosphate concentration of the low nutrient medium was approximately 0.3 µmol/L [20] and that of the high nutrient media approximately 1.8 µmol/L (based on approx. 3.5% phosphate in the yeast extract). While this amount of phosphate would be expected to affect reaction orders and rate constants [19] it is not more than observed at the ALOHA-HOT site used for previous work [5] (approximately 2–3 µmol/L between 500 and 1000 m, http://hahana.soest.hawaii.edu). Thus, the amount of phosphate used in the high nutrient treatments was similar to that observed when *in situ* shallow water dissolution has been observed in the twilight zone.

It has previously been suggested that shallow water calcite dissolution is both common and significant [2]. The process suggested most likely for this dissolution was the development of corrosive microenvironments by respiring microorganisms. Other work [4] has indicated that apparent anomalies in oceanic water chemistry may be explained without the occurrence of significant shallow water dissolution, but the direct observations of physical changes to carbonate substrates incubated in the upper ocean [5], [21] or from shells collected from this zone [8] are thus far unexplained.

Troy et al.[5] conducted an in situ dissolution experiment in the upper ocean (0–1000 m), on non-biogenic calcite, and found significant changes to the surface topography of calcite pieces incubated at depths of 0 to 1000 m for 3 days. There are several inconsistencies in these observations that warrant further discussion and led to our direct assessments of the proposed mechanism. Firstly, the micrographs presented by previous authors have been devoid of bacteria. Although Milliman et al., (1999) [2] state that bacteria were directly observed in the work of Troy et al. (1995) [5], Troy et al. [21] actually states the opposite: “All of the SEM images failed to reveal any attached microbes” (p. 125). Secondly, the previous work [5], [21] used non-biogenic carbonate; a necessity when considering the high precision of their chosen method (atomic force microscopy), but a factor that needs to be considered further. It has been suggested that degradation of carbonate shells proceeds mainly via degradation of the organic matter within the shell matrix, followed by disintegration of the shell as a whole [11]. This is clearly not possible when no organic material is present. The change in surface topography reported also occurred over the whole substrate, which is in contrast to previously reported bacterially mediated dissolution of carbonate shells in sediments [22], visible as distinct zones of dissolution thought to coincide with observed bacterial colonies. In fact, no similar aggregated dissolution patterns have, to our knowledge, been observed on planktonic samples. Finally, it should be noted that the dissolution kinetics of different calcium carbonate materials (aragonite, high and low Mg calcite) differ and that form of the carbonate is important in determining Ω and therefore dissolution [10], [19]. Biogenic carbonates comprise both aragonite and calcite [23] and may differ in their potential contribution to shallow water dissolution depending on their specific composition.

Schiebel et al., [8] suggest that the dissolution is driven by bacterial metabolism within the shell (i.e., a high CO_2_ environment builds up within the shells' chambers because of bacterial degradation of foraminiferal cytoplasm). Intuitively this is more likely, but if true there should be a correlation between the disappearance of cytoplasm and dissolution. No such correlation was observed; in fact, Schiebel et al. [8] specifically reported that shell weight loss was observed to be independent of cytoplasm loss. They also do not address the limitations to aerobic respiration that would be induced by the same transport limitations they use to explain the formation of an acidic micro-environment within the shell chambers. The dissolution of shells from the inside out also does not explain Troy et al.'s [5] results: they used flat carbonate pieces.

Finally, if dissolution is dependent on microbial metabolism it is therefore dependent on time for metabolism. If such an assumption were true then the depth of the zone of dissolution would be dependent on the particle's sinking rate. It may therefore be expected that heavier, faster sinking particles would continue to dissolve over a larger depth interval than lighter, slower particles. Instead, dissolution was observed only between the depths of 500–1000 m, with no dependency on sinking rate. While the above studies provide qualitative evidence for the occurrence of shallow water dissolution, they do not resolve the possible mechanisms.

We investigated the proposed mechanism of dissolution with direct, although not *in situ*, techniques. We employed two methods, one chemical and one visual, to monitor biogenic carbonate dissolution in two shell types (foraminifera and oysters). Microsensors have been employed in a wide variety of calcification studies (e.g., [15], [24], [25], [26]) and have proved a reliable means to detect both calcium precipitation and dissolution. With this direct technique we showed the absence of microenvironments for O_2_, Ca^2+^ and pH at the shell surface. The shell surface chemistry was not significantly different from the surrounding seawater. We were unable to detect bacterially mediated dissolution of our substrates, despite their heavy colonization by bacteria and the formation of EPS, unless we artificially reduced the overlying water calcite saturation state to Ω<1. Despite the rather high natural variation in shell architecture (leading to high within treatment variation) we were able to analyse changes to shell architecture both quantitatively, via CLSM surface topography reconstruction and roughness measures, and qualitatively, via SEM imaging. Despite being able to detect and distinguish between changes induced artificially to shells, by the addition of gluteraldehyde, which both decalcifies carbonate materials and fixes proteins [11] or bleach, which has the opposite effect, we were unable to detect any effect of the presence of bacteria on shell architecture.

The ability of microbial metabolism to generate corrosive microenvironments is dependent upon diffusive limitation under EPS causing the build-up of metabolic products (e.g., CO_2_) able to lower pH. Such diffusional limitation has been previously reported [24]. Metabolic establishment of bacterial micro-environments has also previously been investigated using a modeling approach, validated by measurements, [25], [26] in copepod guts and sinking aggregates, respectively. These studies conclude that microenvironments of low pH do not develop on sedimenting particles and, therefore, imply that this suggested mechanism for calcite dissolution is unlikely.

We, therefore, find the likelihood that the metabolism of colonizing bacteria causes significant dissolution of sedimenting, biogenic carbonates to be very low for several reasons:

1. we failed to observe both chemically and visually, direct bacterial dissolution of carbonate substrates, despite their heavy bacterial colonization, and
2. previous work has shown that oxygen in sinking particles is very close to that of the surrounding seawater [26], and that pH differs less than 0.05 units [27], thus no metabolically driven acid microenvironment develops
3. the time for development of bacterial colonies, and subsequently microenvironments, in actively sinking particles is short (days) and we failed to see dissolution after several weeks,
4. dissolution is reported only from a narrow depth range (700–1000 m), which is not dependent upon the sinking rate of the shells,
5. there is no reported correlation between the disappearance of foraminiferal cytoplasm and shell dissolution, as would be expected if its degradation was fuelling the buildup of an internal, corrosive microenvironment,
6. as discussed above, there have recently been put forward other explanations (e.g., [4], [6] for the water chemistry indicators of shallow water dissolution.

There is much debate regarding the occurrence and extent of shallow water carbonate dissolution. Previous studies have addressed the chemical anomalies that have lead to the assumption of shallow water dissolution via *in situ* assessment of sinking particles and via modeling approaches. Herein we have, for the first time, addressed the actual mechanism suggested responsible for shallow water dissolution: the development of microbially controlled corrosive microenvironments. Using direct methods, we found no evidence that such a mechanism exists, and while our findings do not help explain past qualitative observations of shallow water dissolution, they do indicate that the microbially controlled mechanisms suggested for the occurrence of dissolution are unlikely to contribute significantly to the process.

## Supporting Information

Figure S1 **Scanning electron micrographs of oyster shells treated with bleach (upper) and gluteraldehyde (lower).** Bleach removes roves the shell's organic structure, leaving the mineral component in tact, while gluteraldehyde dissolves the mineral structure (Glover and Kidwell 1993). Visual differences between the two treatments are evident.
(TIF)

Figure S2 **Confocal laser scanning microscope images of shells treated with gluteraldehyde (upper) and bleach (middle) and untreated shells (lower).** Visualdifferences in shell surface architecture are apparent.
(TIF)

Figure S3 **Zeiss surface topography (see** **methods** **) of untreated shell surface and the surface of shells treated with glutereraldehyde and with bleach.** The surfaces of untreated shells are relatively uniform, those of the gluteraldehyde treated shells are large, lower frequency irregularities, while those of the bleach treated shells show smaller, high frequency changes. The surface topography measurements were able to both discern changes to the shell surface and to differentiate by changes induced by alterations to the shell's organic structure (bleach) or its mineral structure (gluteraldhyde).
(TIF)

Figure S4 **Representative calcium microelectrode profile above shell surface in artificial seawater treatment (circles) and after the addition of acid (HCl) to induce dissolution (triangles).** The pH was reduced to 7.1.
(TIF)
