# Supplementary figures and images for: Dissolution of Calcite in the Twilight Zone: Bacterial Control of Dissolution of Sinking Planktonic Carbonates Is Unlikely
Source: PLoS One. 2011 Nov 15;6(11):e26404. doi: 10.1371/journal.pone.0026404 (PMC3216930; doi:10.1371/journal.pone.0026404)

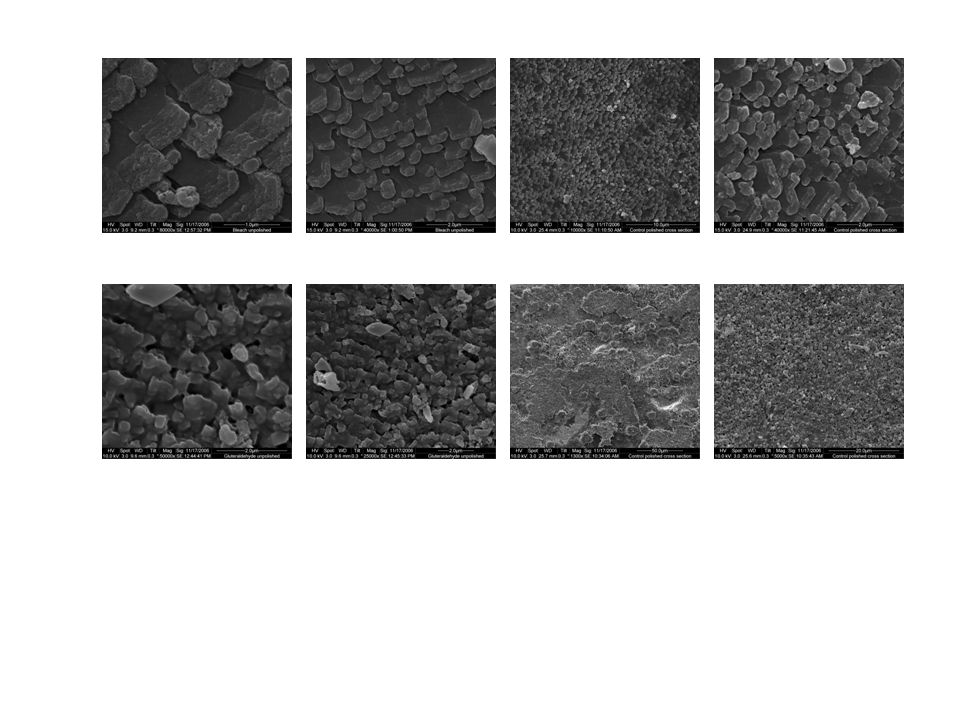

Supplement: Figure S1 — Scanning electron micrographs of oyster shells treated with bleach (upper) and gluteraldehyde (lower). Bleach removes roves the shell's organic structure, leaving the mineral component in tact, while gluteraldehyde dissolves the mineral structure (Glover and Kidwell 1993). Visual differences between the two treatments are evident. (TIF) [file pone.0026404.s001.tif]

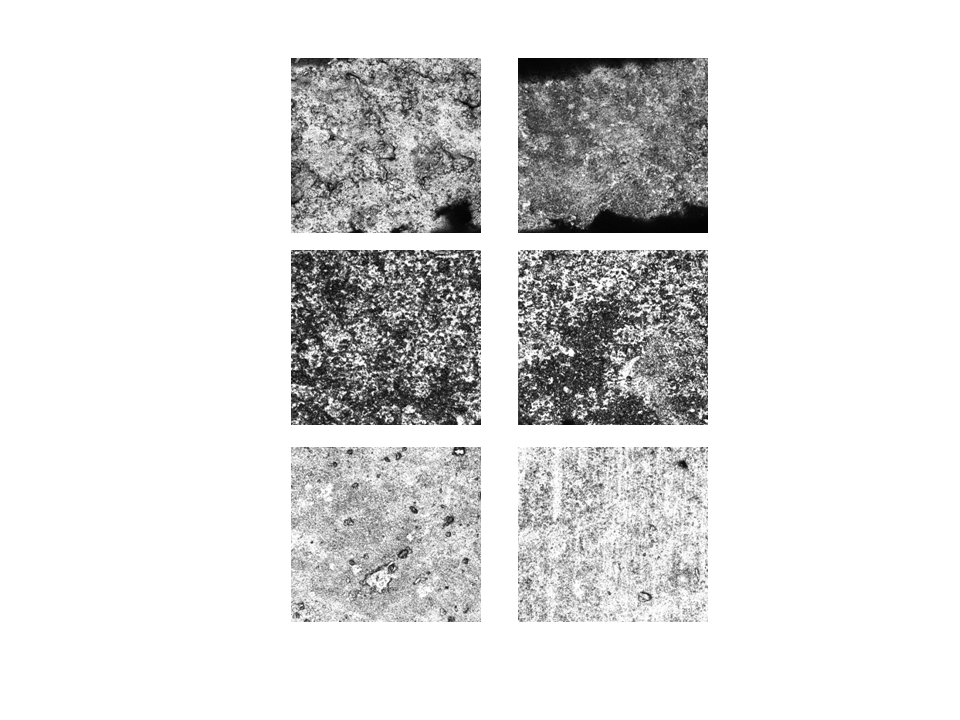

Supplement: Figure S2 — Confocal laser scanning microscope images of shells treated with gluteraldehyde (upper) and bleach (middle) and untreated shells (lower). Visualdifferences in shell surface architecture are apparent. (TIF) [file pone.0026404.s002.tif]

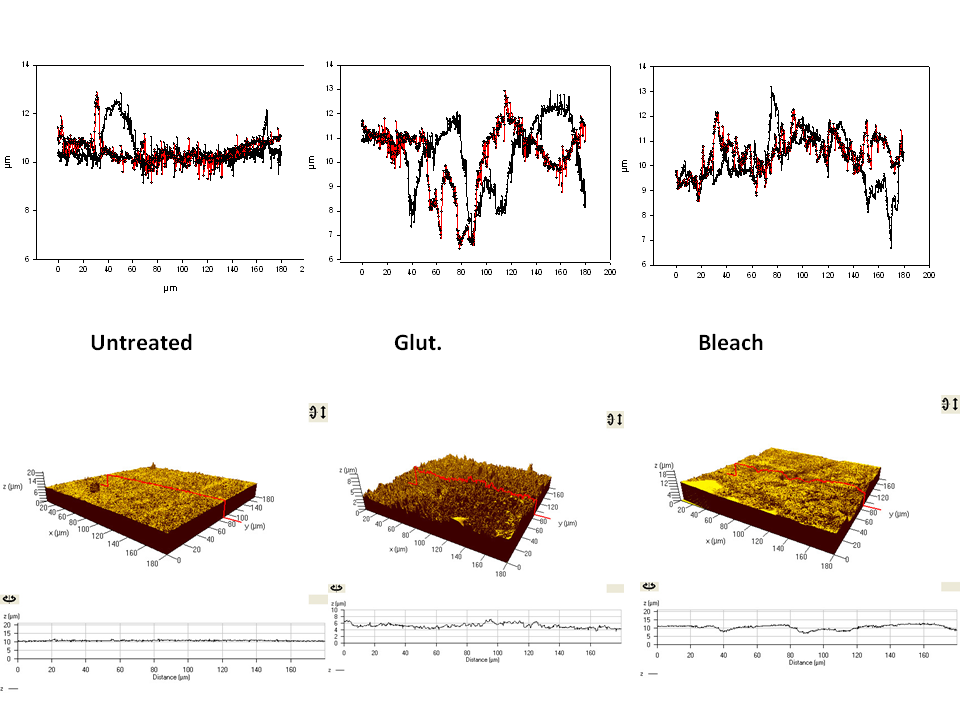

Supplement: Figure S3 — Zeiss surface topography (see methods ) of untreated shell surface and the surface of shells treated with glutereraldehyde and with bleach. The surfaces of untreated shells are relatively uniform, those of the gluteraldehyde treated shells are large, lower frequency irregularities, while those of the bleach treated shells show smaller, high frequency changes. The surface topography measurements were able to both discern changes to the shell surface and to differentiate by changes induced by alterations to the shell's organic structure (bleach) or its mineral structure (gluteraldhyde). (TIF) [file pone.0026404.s003.tif]

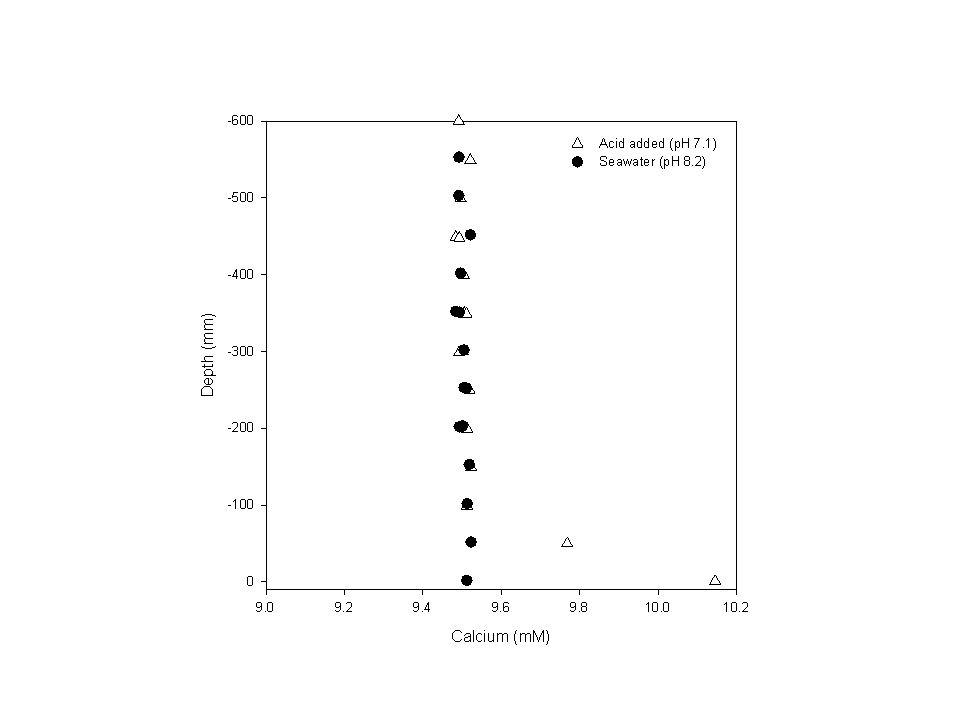

Supplement: Figure S4 — Representative calcium microelectrode profile above shell surface in artificial seawater treatment (circles) and after the addition of acid (HCl) to induce dissolution (triangles). The pH was reduced to 7.1. (TIF) [file pone.0026404.s004.tif]
